# Ancient Indian perspectives and practices of mental well-being

**DOI:** 10.3389/fpsyg.2025.1616802

**Published:** 2025-06-03

**Authors:** Ruchi Bhati, Mitu Mandal, Tripti Singh

**Affiliations:** ^1^Department of Humanities and Social Sciences, Motilal Nehru National Institute of Technology Allahabad, Prayagraj, India; ^2^School of Management studies, Motilal Nehru National Institute of Technology Allahabad, Prayagraj, India

**Keywords:** ancient Indian texts, mental well-being, self-help techniques, ancient psychological practices, Vedas, Upanishads, Bhagavad Gita, Ayurveda

## Abstract

In today’s fast-paced world, mental health issues are rising due to lifestyle changes, social media use, workplace burnout, and geopolitical events. Despite the urgent need to address the concern, mental health remains stigmatized, inaccessible, and undertreated. Contemporary mental health focuses more on symptoms rather than overall mental well-being. The holistic nature of ancient Indian mental health concepts and paradigms includes elements that contemporary mental health literature has overlooked. Indian texts like the Vedas, Upanishads, Bhagavad Gita, and Ayurveda make significant mention of mental health and provide applications of the psychological remedies to real-world circumstances. This study aims to explore and streamline the diverse practices and techniques buried in ancient Indian texts to offer evidence-based, real-world self-help techniques for overall mental well-being.

## 1 Introduction

A state of mental well-being is characterized by an individual’s ability to manage everyday stressors, work efficiently, reach their full potential, and give back to their community. According to WHO (World Health Organization (WHO), 2014), it encompasses emotional equilibrium, resilience in the face of adversity, life satisfaction, and a sense of purpose. According to VanderWeele (VanderWeele, 2021), mental well-being is a multifaceted concept that encompasses elements of psychological functioning, social connections, happiness, and purpose. This growing knowledge emphasizes a change from only treating mental illness to encouraging psychological well-being and adaptability to contemporary difficulties.

In today’s world, mental health has become a significant concern due to the rise in anxiety, depression, and stress-related illnesses. Globally, mental health disorders have significantly increased, according to the World Health Organization (WHO), with depression ranking among the leading causes of disability (World Health Organization, 2021). Social media influence, lifestyle changes, workplace burnout, and sociopolitical uncertainties are examples of modern stressors (Kumar and Nayar, 2020). Because of these problems, there is an excessive dependence on psychiatric drugs, which, although effective, frequently have drawbacks and restrictions (Smith et al., 2019). Despite being evidence-based and popular, contemporary psychological therapies like dialectical behavior therapy (DBT) and cognitive behavioral therapy (CBT) prioritize symptom management over overall well-being (Hayes and Hofmann, 2021). Furthermore, accessibility is still an issue since many people cannot afford treatment or are stigmatized for seeking professional assistance (Patel et al., 2018).

Ancient Indian traditions offer a holistic approach to mental health that integrates physical, mental, and spiritual well-being. The Vedas, Upanishads, Bhagavad Gita, and Ayurveda are a few examples of ancient texts that offer advice on living a balanced life, fostering mental peace, and dealing with psychological problems. The Atharva Veda, for instance, talks about the calming effects of chants and healing sounds, which are now comparable to modern sound therapy and mindfulness-based stress reduction (MBSR) methods (Sharma, 2021). Rich and ancient on the one hand and contemporary on the other, the knowledge treasures are buried throughout India’s many facets. One of the earliest instances of research in this area is the work conducted in India on mental health. Historical records indicate that psychological knowledge and mental health treatment existed more than 3,000 years ago. While Freud is credited with founding psychiatry, his theories date back less than 200 years, and his influence on the field began in the West around 1880 AD. Chanting the age-old prayers, one requests the blessings of treating “Aadhi,” or mental illness, before “Vyadhi,” or physical illness. Asking about mental health before physical health demonstrates how much more important mental health is than physical health. The Rigveda is the earliest piece of Indian literature that links mental health and illness to mythological and religious themes (Balodhi, 1987). There are countless references to mental health and illness in the post-Vedic literature, including the “Brahamanas,” “Tantras,” “Ramayana,” “Mahabharata,” and “Puranas.”

Over the past few decades, there has been an increasing awareness that utilizing India’s rich philosophical and religious traditions is essential to improving the practice of psychiatry—Bangalore’s Prof. N. C. Surya was among the first minds to bring this to their notice (Collins et al., 1996). Professor N. S. Vahia from Bombay was another trailblazer, who through numerous articles, raised awareness of the benefits of using yoga to treat neurotic and India’s psychosomatic illnesses (Vahia et al., 1973). Professor A. Venkoba Rao has written exquisitely about Srimad Bhagwad Gita’s therapeutic value several times in order to comprehend how the mind works (Rao, 1980; Rao, 2003).

The current study aims to gain insight into mental well-being from the ancient Indian perspective. The study investigates the conceptual linkage between ancient Indian texts, such as the Vedas, Upanishads, Gita, and Ayurveda, and foundational principles in mental health, including emotional regulation, cognitive restructuring, and the identification of causative factors of mental illness. It delves into exploring and streamlining the diverse application-based practices and perspectives on mental well-being available in the ancient Indian texts. The holistic nature of ancient Indian mental health concepts and paradigms includes elements that contemporary mental health literature has overlooked. The latter can benefit from borrowing, studying, and using them in their textbooks. The basic entity, the mind, is neglected in favor of biological aspects of psychological phenomena in the current trend of mental health research. This partisan bias must be corrected (Shamasundar, 2008).

## 2 Ancient Indian texts

### 2.1 The Vedas

The Vedic tradition, which has its roots in ancient India, is a vast body of knowledge that includes spiritual practices, philosophies, hymns, and rituals. The mind, body, and spirit are said to be aligned through the use of Vedic mantras, which are thought to possess spiritual and vibrational power. Mantra chanting is believed to improve mindfulness, soothe the neurological system, and control breathing patterns. Researchers such as Frawley (Frawley, 2012) contend that the acoustic waves of mantras correspond with specific brainwave patterns, potentially promoting a meditative state and mitigating psychological stress. According to the Rigveda and Upanishads, mantras’ rhythmic recitation can help dissolve mental clutter and foster inner peace (Kumar, 2015). The concepts of psychoneuroimmunology and vibrational medicine serve as the theoretical foundation for the application of Vedic mantras in mental health. According to the vibrational theory, different sound frequencies can affect psychological states by changing brainwave patterns (Mahajan et al., 2021). Mantras may also promote positive changes in neural pathways linked to mood regulation and stress response by facilitating neuroplasticity (Nair and Telles, 2022). Understanding the use of Vedic mantras in contemporary mental health practices has gained popularity in recent years, especially for the treatment of stress, anxiety, depression, and general mental health issues.

### 2.2 The Upanishads

The philosophical writings of Hinduism, the Upanishads, offer significant perspectives on emotional and mental health. These texts highlight self-reflection, self-control, self-inquiry, and detachment as essential techniques for achieving inner peace and clarity. Current research backs up these ideas, emphasizing how well they work to lower stress, improve emotional control, and enhance cognitive function.

One of the fundamental practices delineated in the Upanishads is Atma Vichara, or self-inquiry, which advocates for individuals to contemplate their intrinsic essence beyond the confines of material existence [Brihadaranyaka Upanishad 4.4. (Radhakrishnan, 1994)] Empirical evidence indicates that individuals who consistently engage in self-inquiry attain enhanced self-awareness, emotional clarity, and diminished stress responses (Brown and Ryan, 2003). Another pivotal notion is Dama, which pertains to the regulation of the mind, accomplished through the control of breath and the practice of mindfulness [Katha Upanishad 1.3.3–6 (Radhakrishnan, 1994)]. This is comparable to contemporary mindfulness-based stress reduction (MBSR) practices, which have been shown to enhance emotional stability and cognitive flexibility, two qualities that are central to Upanishadic teachings (Tang et al., 2015). The Upanishads also elaborate on Ahamkara, or the concept of non-attachment to the ego, which cultivates a sense of humility and emotional stability [Mundaka Upanishad 3.1.1 (Radhakrishnan, 1994)]. According to research on ego dissolution and emotional health, feelings of compassion, thankfulness, and mental stability rise when one’s excessive identification with the ego decreases (Niemiec, 2019).

The mental health practices delineated in the Upanishads, such as self-inquiry, mindfulness, non-attachment, and emotional equilibrium, possess significant relevance in contemporary psychology, therapy, workplace wellness, and personal growth. These age-old doctrines correspond with empirically substantiated mental health strategies, thereby affirming their enduring significance.

### 2.3 The Bhagavad Gita

The Bhagavad Gita is a dialogue from the epic Mahabharata between the Pandava prince Arjuna and his charioteer Krishna, and explains many psychotherapy concepts (Bhatia et al., 2013). The Bhagavad Gita places great emphasis on achieving sthitaprajna, which means to be settled in a state of prudent temperance or equanimity. One can reach this state by using niṣkama karma, or dispassionate action, to effectively manage thoughts, emotions, and desires (Singh and Raina, 2015). Using this fundamental principle of the Bhagavad Gita, Bhawuk (Bhawuk, 2011) illustrates the complex interweaving of thoughts, cognition, and behavior and how these interactions impact human welfare. He says that when an embodied person gets identified with an objective, a powerful drive to accomplish the objective arises. At this point, the person takes action to accomplish the goal, which may have a wide range of emotional repercussions. Since desires are viewed as “fire that is never satiated,” a person is always entangled and burdened by feelings and thoughts [Bhagavad Gita, Verse 3.39 (Prabhupada, 1972)]. This fundamentally entails the execution of every endeavor and ambition with fervor while simultaneously refraining from personal expectations or entanglements. It’s about letting go of attachment to the outcome and staying in a non-attachment state (anasakti). We can attain this through meditation and introspection by critically analyzing and questioning our desires and how they impact us. The idea of non-attachment, or anasakti, is related to Peterson and Seligman’s model of character strengths and virtues in positive psychology (Peterson and Seligman, 2004).

The Bhagavad Gita is a valuable resource on mental health, offering techniques for rewiring the brain, managing stress, and controlling emotions. There is potential to improve the efficacy of mental health interventions by incorporating these antiquated teachings into contemporary therapeutic practices, especially in settings that are culturally congruent.

### 2.4 The Ayurveda

Ayurveda, which means “science of life,” is an ancient science that has its roots in the Atharvaveda. While vedantic treatises also mention Ayurvedic principles, the two most famous written accounts of Ayurveda are the Charak Samhita (1,400 BC) and the Sushrut Samhita (1,500 BC). These two timeless works delineate mental illnesses, personality types based on the trigunas (satva, raj, and tam) and tridoshas (the three bodily humors, vata, pitta, and kapha) (Gautam, 1999). Ayurveda holds that improving Sattva and achieving a balance between Rajas and Tamas are necessary for mental well-being (Rao, 2011). The causes of mental illnesses are discussed in the chapter on manasrog. Mental disorders are frequently associated with Vata imbalance (Singh, 2009). According to Ayurveda, maintaining the right balance between the three humors (Doshas) and the five elements (Bhutas) is the key to good health. Ojas, known as the essence of life, is thought to be essential for immunity and mental toughness. For mental health, Ayurvedic practices that support Ojas include meditation, a balanced diet, and enough sleep (Lad, 2002). Balance exists on several levels, including the physiological, psychological, and lastly spiritual, which is a blissful state where peace is the ultimate objective (Verhagen et al., 2010).

By contrasting the clinical conditions found in Ayurveda with those found in the International Classification of Diseases, Dube has conducted a systematic investigation into the nosology and treatment of mental illness in Ayurveda (Dube, 1978). When it comes to mental diseases, the 16 types of personality constitutions are likely to experience illnesses that can be correlated with any of the 16 different mental disorders. Dube has detailed how Ayurvedic diagnostic categories correlate with comparable ICD diagnoses. Devgraheet and kafaj, for instance, are comparable to simple schizophrenia; vataj, gandharvagraheet, is comparable to mania; pittagraheet, manasdukh, is comparable to depression; pittaj and rakshasgraheet, to catatonic schizophrenia; paishachgraheet, to hebephrenic schizophrenia; shaponmatt, to hysteria; daityagraheet, to antisocial personality; sarpgraheet, to organic psychosis; nishd, to mental deficiency; vaital, kushmand, and vishjonmad, are comparable to organic psychoses; atatvavibhinesh, to senile and pre-senile dementia; and aukiran, to chronic psychoses.

## 3 Linkages of ancient Indian scriptures and practices with mental well-being

**Figure 1:**
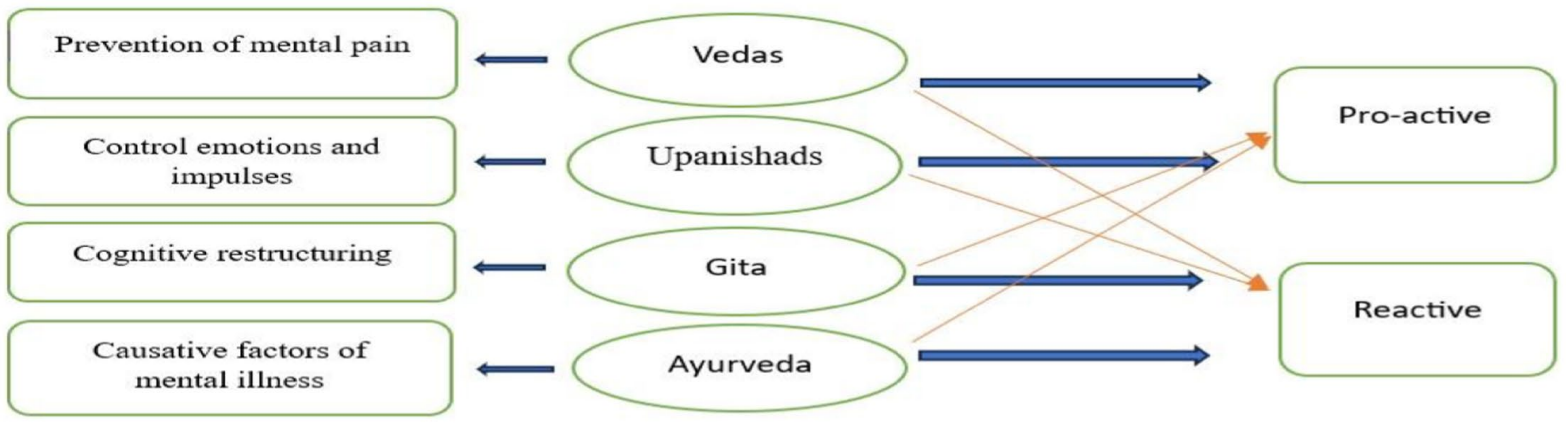
Linkage of mental health concepts to ancient Indian texts and catergorisation into proactive and reactive approaches.

In Figure 1, mental health concepts are linked to ancient Indian texts and categorized into proactive and reactive approaches. The left column indicates the key mental health strategies associated with the ancient Indian Texts. Vedas majorly focus on the prevention of mental pain (Avasthi et al., 2013). It provides techniques for preventing mental distress before it occurs. Mental health practices in the Upanishads majorly focus on controlling emotions and impulses, indicating strategies for controlling emotional reactions (Gautam, 1999). Gita majorly emphasizes cognitive restructuring, which is modifying one’s mental state by altering one’s thought patterns, as illustrated in “Sankhya Yoga” in chapter 2 of Bhagavad Gita. Lord Krishna’s cognitive restructuring of Arjuna through the Yoga of Jnana, Bhakti, and Karma offers a comprehensive framework that promotes psychological conflict resolution (Rao, 2009). Ayurvedic literature provides detailed explanations of the manas (mind), Mental faculties (causative factors) like “Shoka” and “Krodh,” causes, cures, and prevention of Manasa vyadhi (mental illness), with a focus on mental health (Khayamali et al., 2024).

In the right column, Blue Arrows (Thicker) highlight the text’s primary focus on a specific strategy. Red Arrows (Thinner) indicate that the approach has less influence or secondary impact on a specific strategy. A major proportion of the Vedas is pro-active as it places a strong emphasis on upholding equilibrium and avoiding mental suffering by encouraging moral behavior, introspection, and harmony with the natural world (Sharma, 2013). The approach of the Upanishads is both proactive and reactive. Majorly, it’s pro-active as detachment from desires, ego, and material pleasures is recommended by the Upanishads in order to achieve emotional mastery. The Upanishads are primarily a proactive tool for fostering self-awareness, but they also provide a reactive approach by addressing suffering through self-realization (Paranjpe, 1998). The approach of the Gita is mainly reactive in nature, as the central theme of the Gita is changing one’s perspective by accepting responsibility (Dharma) and distancing oneself from results (Karma Yoga). It helps Arjuna move from hopelessness to action by guiding him through his emotional turmoil (Rao et al., 2008). By promoting a balanced mind, the Gita also teaches proactive mental resilience (Sthitaprajna). The nature of Ayurveda is also majorly reactive in nature, as the main medical focus of Ayurveda is determining the underlying causes of mental disorders (such as a Dosha imbalance) and recommending holistic therapies that incorporate lifestyle, food, herbs, and mental exercises (Patel, 2018). Therefore, it could be inferred that the Vedas and Upanishads place more emphasis on proactive mental health techniques that lessen emotional distress. Gita guides emotional transformation by fusing Cognitive Restructuring with both Proactive and Reactive approaches. Ayurveda is primarily a reactive system that focuses on identifying and treating mental health conditions.

## 4 Application-based techniques and practices in ancient Indian texts

Table 1 provides an overview of the various ways that the Vedas, Upanishads, the Bhagavad Gita, and Ayurveda, among other Indian texts, address mental and emotional health using useful self-help methods. Every intervention is intrinsically linked to a scriptural verse, demonstrating the age-old, timeless wisdom that underpins contemporary mental health techniques like self-reflection, breathwork, and mindfulness.

**Table 1:** Overview of the self-help techniques present in ancient Indian texts.

S. No.	Scriptures	Intervention	Description	Target	Verse
1	Vedas	Svadhyaya	Self-reflection, Introspection	Reduction in inner conflict and emotional regulation	Rig Veda 1.164.33
		Nadi Sodhana or Ujjayi pranayama	Controlling and regulating the breath	Mental Clarity, reduction in stress	Rig Veda 10.53 and Atharvaveda 4.33.4
		Mantra Chanting	Chanting with intent focus	Lessen anxiety and cleanse the mind.	Rig Veda 1.164.39
2	Upanishads	Atma Vichara	Regularly reflect on one’s true nature (Self inquiry)	Clarity and confidence	Brihadaranyaka Upanishad (4.4.5)
		Dama	Mind control through breathing exercises or mindfulness.	improves concentration and lessens overthinking	Katha Upanishad 1.3.3–6
		Ahamkara	Non-Attachment to Ego	fosters emotional equilibrium and humility	Mundaka Upanishad 3.1.1
3	Gita	Dhayana	Meditation and Mindfulness	Mental clarity and Self-awareness	Bhagavad Gita 6.12–6.13
		Stithprajna	Steady Intellect	Equanimity of the mind	Bhagavad Gita 2.54
		Vairagya	Detachment and self-awareness	lessen tension and enhance mental acuity	Bhagavad Gita 2.47
		Samatva	Calm in the face of adversity	Balanced and composed mind	Bhagavad Gita 2.14, 2.70
4	Ayurveda	Panchakarma	Detoxification and purification of the body	Mental and emotional well-being	Charak Samhita, sutrasthana 11.3
		Rasyana (Bhrami, Ashwagandha, jatamansi)	Herbal and natural remedies	Enhances memory, lowers stress, and anxiety	Charak Samhita, Sutrasthana 24.52
		Abhyanga	Daily oil message	Calms the mind and grounds emotions.	Ashtanga Hridayam 2.8
		Balancing Agni	Consumption of triphala and warm water	enhances clarity and removes mental fog	Charaka Samhita 15.3

According to the Vedas, self-reflection, or Svadhyaya, promotes introspection to lessen internal conflict and foster emotional control [Rig Veda 1.164.33 (Prabhupada, 2003)]. Breathing exercises like Ujjayi Pranayama and Nadi Shodhana are emphasized for their ability to improve mental clarity and calm the nervous system [Rig Veda 10.53 (Prabhupada, 2003), Atharvaveda 4.33.4 (Prabhupada, 2002)]. This view is supported by recent research on the benefits of breathwork for reducing stress (Saoji et al., 2019). The Rig Veda 1.164.39 also suggests mantra chanting as a way to clear the mind and reduce anxiety (Prabhupada, 2003), which is consistent with recent research on the calming effects of repetitive sounds during meditation (Bernardi et al., 2001). fMRI results while participants chanted “OM” vs. rest, showed the activation of brain areas such as the amygdala, thalamus, insula, and cingulate cortex, indicating a vagal afferent pathway akin to vagus nerve stimulation, which is known to alleviate depression and stress (Kalyani et al., 2011). EEG patterns showed that significant increase in theta2 and alpha1 frontal, parietal, and frontal–parietal coherence while listening to Vedic recitation compared to practicing Transcendental Meditation (Travis et al., 2017).

The Upanishads support Atma Vichara, or self-inquiry, which is similar to contemporary cognitive behavioral techniques that foster self-awareness (Neff, 2003). It encourages regular reflection on one’s true nature to develop clarity and confidence [Brihadaranyaka Upanishad 4.4.5 (Radhakrishnan, 1994)]. According to studies on mindfulness’s ability to enhance cognitive function (Zeidan et al., 2010), Dama, or mind control through breathing and mindfulness, is known to increase focus and decrease overthinking [Katha Upanishad 1.3.3–6 (Radhakrishnan, 1994)]. Furthermore, according to Mundaka Upanishad 3.1.1 (Radhakrishnan, 1994), the idea of Ahamkara (non-attachment to ego) promotes emotional balance and humility, which is consistent with contemporary psychological theories on ego dissolution and emotional health (Niemiec, 2019).

The Bhagavad Gita highlights techniques such as Dhyana (meditation) to develop self-awareness and mental clarity [Bhagavad Gita 6.12–6.13 (Prabhupada, 1972)], which is consistent with the extensive research showing that meditation helps people become more emotionally resilient and less anxious (Goyal et al., 2014). According to the Bhagavad Gita (2.54), the idea of Stithaprajna (steady intellect)—maintaining mental composure in the face of adversity—echoes contemporary ideas of emotional control (Gross, 2002). Additionally, Vairagya (detachment) and Samatva (emotional balance) encourage mental toughness and poise in the face of hardship [Bhagavad Gita 2.47, 2.14, 2.70 (Prabhupada, 1972)], which is similar to contemporary therapeutic approaches that emphasize commitment and acceptance (Hayes et al., 2006). The Gita’s teachings align with modern psychology, offering potential mental health benefits, but empirical studies are sparse (Dhillon, 2023). In a study, the viability and effectiveness of a psychospiritual intervention based on Yin Yoga and the Bhagavad Gita were investigated in a Turkish community. The mixed-method approach showed that participants’ mental health improved, proving the cross-cultural applicability of Gita-based teachings in therapeutic contexts (Akartuna et al., 2025).

Ayurveda uses physical treatments to support these mental practices. By removing toxins that harm both physical and mental health, panchakarma, a detoxification treatment, promotes mental and emotional well-being [Charaka Samhita, Sutrasthana 11.3 (Srikantha Murthy, 2001)]. According to recent research on adaptogenic herbs, rasayana herbs like Brahmi, ashwagandha, and jatamansi are well known for improving memory, lowering stress, and boosting cognitive function [Charaka Samhita, Sutrasthana 24.52 (Srikantha Murthy, 2001; Singh et al., 2011)]. With the use of Ashwagandha significant drops in anxiety and depression scores in adults were observed in a randomized controlled experiment (RCT) when compared to a placebo (Chandrasekhar et al., 2012). improvements in cognitive performance in healthy persons who received 300 mg of standardized brahmi extract daily for 12 weeks were recorded (Stough et al., 2001). Last but not least, research showing the role of therapeutic touch in lowering cortisol levels and improving relaxation (Field, 2016) supports the recommendation that Abhyanga, a daily oil massage, be used to ground emotions and promote calmness [Ashtanga Hridayam 2.8 (Vagbhatacharya, 2001)]. A thorough, all-encompassing approach to mental health and emotional resilience is provided by these ageless self-help techniques from Indian texts, which are backed by both traditional knowledge and contemporary studies. These methods, which have their roots in spiritual and philosophical knowledge, complement contemporary psychological approaches to stress reduction, mindfulness, and introspection.

## 5 Conclusion

The ageless wisdom found in Indian scriptures is becoming more and more applicable to contemporary mental health procedures. A thorough, all-encompassing approach to mental health and emotional resilience is provided by these ageless self-help techniques from Indian texts, which are backed by both traditional knowledge and contemporary studies. Evidence for the benefits of Vedic mantras, practices of Upanishads, techniques in Bhagavad Gita, and ayurvedic remedies on anxiety, stress, depression, and cognitive function suggests that they could be used in addition to other treatments to improve mental health. While the available research is encouraging, more thorough investigations are required to properly determine the effectiveness and mechanisms of these early interventions. By using larger, more diverse samples and standardized intervention protocols, future research should strive to address the methodological limitations of previous studies. A more thorough understanding of the effects of various ancient Indian mental health practices can be obtained by incorporating objective physiological and neurological measures.

## Data Availability

The original contributions presented in the study are included in the article/supplementary material, further inquiries can be directed to the corresponding author.
